# Anæsthesia

**Published:** 1864-11

**Authors:** 


### Selections.

ANÆSTHESIA.—There has been much discussion in our medical journals, within the last fifteen years, in relation to the discovery of the anæsthetic properties of sulphuric ether.

Several persons have claimed the honor of the discovery, but it can not belong to them all.

I remember an incident in connection with this matter which I will relate.

During the term of 1843–44, I was attending lectures in the medical department of Harvard University.

Dr. J. C. Warren remarked to the class, at the close of one of his lectures, that there was a person in the building by the name of Horace Wells, from Hartford, Conn., who had, or thought he had, discovered an agent which would relieve or prevent pain in surgical operations. Dr. Warren also said that it would be a great blessing if it should prove true, and advised the class to see Mr. Wells. We met him in another part of the college, where he explained to us the nature of the discovery, and gave it as his opinion that it would alleviate and probably prevent *all* pain in surgical operations. Mr. Wells made some experiments before the class with this new agent, but I was not able to remain and see them at this time. But I did witness some of his experiments in the evening of the same day, in another place, and was highly pleased with the results, and I think the class were greatly interested in his remarks and experiments.

I was a resident of Boston for several years subsequently, and I never heard of any other person making any claim to the discovery, for some two or three years after the above named period.

I have not the least doubt but that we are indebted to Horace Wells for the discovery of this invaluable mitigator of human suffering.

I esteem Dr. Jackson highly for his scientific researches, and have admired the industry of Dr. Morton in calling the attention of the profession and the public generally to this valuable agent, but their efforts to appropriate to themselves the *honor* and pecuniary benefit of Mr. Wells' discovery, must be regarded as a *great wrong*. MASON M. MILES, M. D.

[*Medical and Surgical Reporter.*]

